# Effect of Digital Care Platforms on Quality of Care for Oncological Patients and Barriers and Facilitators for Their Implementation: Systematic Review

**DOI:** 10.2196/28869

**Published:** 2021-09-24

**Authors:** Jana S Hopstaken, Lynn Verweij, Cees J H M van Laarhoven, Nicole M A Blijlevens, Martijn W J Stommel, Rosella P M G Hermens

**Affiliations:** 1 Department of Surgery Radboud Institute for Health Sciences Radboud University Medical Center Nijmegen Netherlands; 2 Department of Hematology Radboud Institute for Health Sciences Radboud University Medical Center Nijmegen Netherlands; 3 Department of IQ Healthcare Radboud University Medical Center Nijmegen Netherlands

**Keywords:** digital care platforms, cancer care, eHealth, telemedicine, health care services, fragmentation of care, health care fragmentation, oncology, quality of care, barriers, facilitators, patient experience

## Abstract

**Background:**

Oncological health care services are challenged by the increasing number of cancer survivors, long-term follow-up care, and fragmentation of care. Digital care platforms are potential tools to deliver affordable, patient-centered oncological care. Previous reviews evaluated only one feature of a digital care platform or did not evaluate the effect on enhancement of information, self-efficacy, continuity of care, or patient- and health care provider–reported experiences. Additionally, they have not focused on the barriers and facilitators for implementation of a digital care platform in oncological care.

**Objective:**

The aim of this systematic review was to collect the best available evidence of the effect of a digital care platform on quality of care parameters such as enhancement of available information, self-efficacy, continuity of care, and patient- and health care provider–reported experiences. Additionally, barriers and facilitators for implementation of digital care platforms were analyzed.

**Methods:**

The PubMed (Medline), Embase, CINAHL, and Cochrane Library databases were searched for the period from January 2000 to May 2020 for studies assessing the effect of a digital care platform on the predefined outcome parameters in oncological patients and studies describing barriers and facilitators for implementation. Synthesis of the results was performed qualitatively. Barriers and facilitators were categorized according to the framework of Grol and Wensing. The Mixed Methods Appraisal Tool was used for critical appraisal of the studies.

**Results:**

Seventeen studies were included for final analysis, comprising 8 clinical studies on the effectiveness of the digital care platform and 13 studies describing barriers and facilitators. Usage of a digital care platform appeared to enhance the availability of information and self-efficacy. There were no data available on the effect of a digital care platform on the continuity of care. However, based on focus group interviews, digital care platforms could potentially improve continuity of care by optimizing the exchange of patient information across institutes. Patient-reported experiences such as satisfaction with the platform were considerably positive. Most barriers for implementation were identified at the professional level, such as the concern for increased workload and unattended release of medical information to patients. Most facilitators were found at the patient and innovation levels, such as improved patient-doctor communication and patient empowerment. There were few barriers and facilitators mentioned at the economic and political levels.

**Conclusions:**

The use of digital care platforms is associated with better quality of care through enhancement of availability of information and increased self-efficacy for oncological patients. The numerous facilitators identified at the patient level illustrate that patients are positive toward a digital care platform. However, despite these favorable results, robust evidence concerning the effectiveness of digital care platforms, especially from high-quality studies, is still lacking. Future studies should therefore aim to further investigate the effectiveness of digital care platforms, and the barriers and facilitators to their implementation at the economic and political levels.

## Introduction

### Background

Over the past few decades, the management of patients with cancer has considerably changed. Owing to earlier detection of cancer and improved treatment strategies, the number of cancer survivors has increased [1]. For this reason, patients with cancer currently require long-term follow-up care, similar to patients with chronic diseases. It is expected that the increased need of follow-up care, in combination with an overall increase of cancer patients due to an aging society, will intensify the use of health care services and increase health care costs [2]. Current health care systems cannot accommodate these increased demands and are deemed unsustainable [3,4]. An additional problem that requires a reevaluation of oncological health care services is the multidisciplinary, and sometimes multi-institutional, aspect of current care. This refers to the fact that cancer patients are usually cared for by multiple health care providers (HCPs), and that, as a result of centralization of complex care to high-volume centers [5], patients may receive parts of their treatment in multiple hospitals [6-9]. These aspects result in the fragmentation of cancer care [10].

The challenges facing cancer care are recognized by the World Health Organization [11] and the Health Program of the European Union [12], and have prompted them to think of different health care structures that enable the delivery of affordable, coordinated, patient-centered oncological care. A solution in restructuring health care for oncological patients could be found in telemedicine or electronic health (eHealth). Moreover, the COVID-19 pandemic, which led to the sudden forced implementation of telemedicine, has demonstrated the potential of telemedicine and eHealth to many patients and doctors [13]. These technologies therefore seem to be indispensable for sustainable care.

For these reasons, the time seems right to deploy eHealth much more widely in oncological care. An updated, accepted definition of eHealth is lacking, but eHealth can be perceived as an umbrella term for all digital communication and information technologies that aid in health care or health care services [14,15]. Over the past decades, multiple eHealth interventions have been developed to support oncological care [16,17]. The eHealth-based intervention of interest in this review is a digital care platform. We define a digital care platform as an eHealth-based tool that aims to increase coordinated and patient-centered care. A digital care platform incorporates several different features, which separately have been the subject of study elsewhere [18-21]. First, digital care platforms may provide patients with information specific to their situation. Second, they can provide an overview of the patients’ personal health records, including appointments, medical results, and correspondence. Third, digital care platforms may also offer direct, secure messaging with HCPs (eg, electronic consultation [e-Consult]). We consider these three characteristics as key features of a digital care platform. Some additional features may include the registration of patient-reported outcomes (PROMs), a patient forum, the possibility to exchange patient-related information between different health care institutes, and enable communication between HCPs and general practitioners.

Studies on the use of eHealth-based tools and their effect on care processes have been previously described for patients with chronic diseases [22,23]. A recent systematic review by Tighe et al [23] indicated that digital platform–like interventions such as self-management tools have a positive effect on physical activity and disease-related quality of life. A systematic review by Kooij et al [22] indicated that digital interventions had positive effects on patient confidence and HCP satisfaction, but that firm conclusions on its clinical effects could not be drawn. Studies on the use of eHealth in oncological care have reported favorable results, as it has been associated with improved patient-provider communication [24]; improved coping with cancer-related symptoms such as fatigue, depression, anxiety, and physical activity [16,25-28]; and improved medication adherence and higher patient satisfaction [20,27]. Some studies have also reported favorable effects on quality of life [29,30]. However, these studies investigated the effect of only one feature of a digital care platform, for instance the registration of PROMs. Previous reviews did not study the digital care platform as a central part of the oncological health care service or the effect of digital care platforms on enhancement of information, continuity of care, or patient- and HCP-reported experiences. The effect on patient self-efficacy has only been studied for one feature of a digital care platform, namely self-management programs [25]. The outcome parameters of interest (ie, enhancing availability of information, self-efficacy, continuity of care, and patient-reported and HCP-reported experiences) can also be placed in the Quality of Care framework provided by the Institute of Medicine [31]. The six domains of quality of care are safe, effective, patient-centered, timely, efficient, and equitable care. Enhancing availability of information is a measure of effective and equitable care, self-efficacy is a measure of patient-centered care, patient-reported and HCP-reported experiences are measures of safe and patient-centered care, and continuity of care is a measure of efficient care and arguably also timely care.

Additionally, we did not identify any existing systematic reviews that focused on the barriers and facilitators that exist for the successful implementation of a digital care platform in oncological care. This is important to assess so as to adequately determine the feasibility of a digital care platform as part of routine oncological care.

### Objectives

The aim of this systematic review was two-fold: (1) to collect the best available evidence of the effect of a digital care platform on quality of care for oncological patients by focusing on enhancement of available information, self-efficacy, continuity of care (including communication), and patient-reported and HCP-reported experiences; and (2) to analyze the currently reported barriers and facilitators for implementation of a digital platform in oncological health care.

## Methods

### Protocol and Registration

This systematic review protocol was registered with the International Prospective Register of Systematic Reviews (PROSPERO; registration number CRD42020199282) [32] and was carried out according to the PRISMA (Preferred Reporting Items for Systematic Review and Meta-Analyses) guidelines [33].

### Search Strategy

The PubMed (Medline), Embase, CINAHL, and Cochrane Library databases were searched for the period from January 1, 2000 until May 27, 2020. Search terms used included “neoplasms” and affiliated terms combined with “patient portal” or “digital care” or “eHealth.” The aim of this search query was to provide search results on literature involving both the effect of a digital care platform on quality of care for oncological patients as well as barriers and facilitators for implementation. The exact search query is shown in Textbox 1.

**Search query.**

(neoplasms [mesh] OR cancer* [tiab] OR tumor* [tiab] OR tumour* [tiab] OR neoplasm* [tiab] OR malignan* [tiab])

AND

(“Patient Portals”[Mesh] OR “Telemedicine” [Mesh] OR Patient portal*[tiab] OR Patient platform*[tiab] OR Patient web portal*[tiab] OR Patient internet portal*[tiab] OR virtual care*[tiab] OR digital care [tiab] OR ehealth [tiab] OR e-health [tiab] OR econsult [tiab] OR e-consult [tiab])

### Eligibility Criteria

Studies of interest included randomized controlled trials (RCTs), prospective studies, and retrospective cohort studies. In addition, qualitative studies in which questionnaires were distributed or stakeholders were interviewed to investigate barriers and facilitators were also included. All studies were required to generate empirical data. Studies not written in English were also screened on the condition that they presented an English abstract. A translator could translate the full text if the abstract seemed to be eligible, thus avoiding a language bias. Systematic and narrative reviews, conference abstracts, and single case reports were excluded.

**Figure 1:**
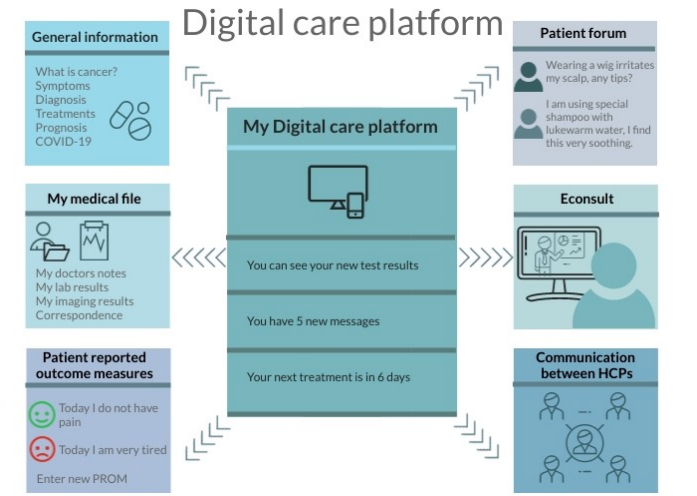
Explanatory illustration of a digital care platform. Studies included in this systematic review were required to investigate a digital care platform with at least two of the following features: (1) general information provision, (2) electronic patient file, or (3) electronic consult (Econsult) with health care providers (HCPs). PROM: patient-reported outcome measure.

For our first objective, to collect the best available evidence of the effect of a digital care platform on quality of care, studies were required to involve oncological patients ≥18 years old. These clinical studies had to assess the effectiveness of a digital care platform. Although we provided our definition of a digital care platform in the Introduction, considering the exploratory stage of research of digital platforms in oncological care, we suspected that there would be a rather limited number of studies investigating such an extensive digital care platform. For this reason, for inclusion in our systematic review, a digital care platform was required to have at least two of the following key features: (1) provide general information concerning the disease as education (eg, symptoms, treatment, follow-up, prognosis); (2) provide patient-specific information concerning their medical file, such as planned appointments, treatments, and lab results; and (3) enable patients to communicate with their physician or specialized nurse via chatting, e-Consult, or email. Figure 1 depicts these and other features of a digital care platform.

Studies that did not involve a digital care platform with at least two of the three above-mentioned features and studies that provided insufficient or vague details concerning the digital intervention were excluded from the analysis to assess our primary objective.

For the second objective, identification of barriers and facilitators for implementation of a digital care platform in oncological care, studies were not required to involve a digital care platform that was already implemented. They could comprise studies that actually implemented a digital care platform and subsequently described the barriers and facilitators for implementation, but they could also comprise studies that identified barriers and facilitators based on a hypothetical discussion with stakeholders. In the latter case, the digital care platform in question was not yet implemented or developed.

### Study Selection

Two separate reviewers (JH and LV) screened the search output on titles and abstracts using Rayyan software [34]. During this screening process, the reviewers were blinded to each other’s decisions. Studies with a contradictory judgment were discussed. In case of a remaining discrepancy, senior reviewers (RH and MS) were asked to arbitrate. The full texts of the selected studies were screened for eligibility. Reference lists of studies that were included after full-text screening were checked for additional eligible studies (snowball method). In cases in which multiple eligible studies reported on the same dataset (≥50% overlap of sample size), we excluded the article with the shortest follow-up.

### Data Extraction and Analysis

Two authors (JH and LV) extracted data using a shared template. These data included: (1) author, year of publication; (2) country; (3) study design; (4) patient population, sample size; (5) platform that was the subject of the study; 6) features of the platform; and (7) the outcome parameters, including enhancement of available information, self-efficacy, continuity of care, and patient- and HCP-reported experiences. If possible, data were pooled; otherwise, synthesis of the results was performed qualitatively.

Barriers and facilitators for implementation of digital care platforms were categorized according to the framework of Grol and Wensing [35]. This framework categorizes barriers and facilitators at six different levels: (1) innovation, which involves advantages in practice, feasibility, credibility, accessibility, and attractiveness; (2) individual professional, which concerns the awareness, knowledge, attitude, motivation to change, and behavioral routines of the involved professionals; (3) patient, which involves knowledge, skills, attitude, and compliance of the patients; (4) social context, which concerns opinion of colleagues, culture of the network, collaboration, and leadership; (5) organizational context, which includes the organization of the care processes, staff, capacities, resources, and structures; and (6) economic and political contexts, which involve regulations, financial arrangements, and policies. The frequency of the barriers and facilitators mentioned, and the quality of the studies were used to prioritize barriers and facilitators.

### Critical Appraisal of Evidence

The Mixed Methods Appraisal Tool (MMAT) version 2018 was used to critically appraise the included studies [36]. The MMAT is designed to help reviewers appraise the quality of empirical studies with different methods. These include quantitative RCTs, quantitative nonrandomized trials, quantitative descriptive studies, as well as qualitative and mixed methods studies. Each category includes five different quality parameters, all requiring to be assessed by answering “yes,” “no,” or “cannot tell” (maximum total score=5). Two authors (JH and LV) independently appraised all studies. In case of disagreement, RH and MS were asked to arbitrate. Studies with a total score of 1-2 were considered to be of low quality, a study with a score of 3 was considered to be of moderate quality, and studies with total scores of 4-5 were considered high-quality studies.

## Results

### Search Results

**Figure 2:**
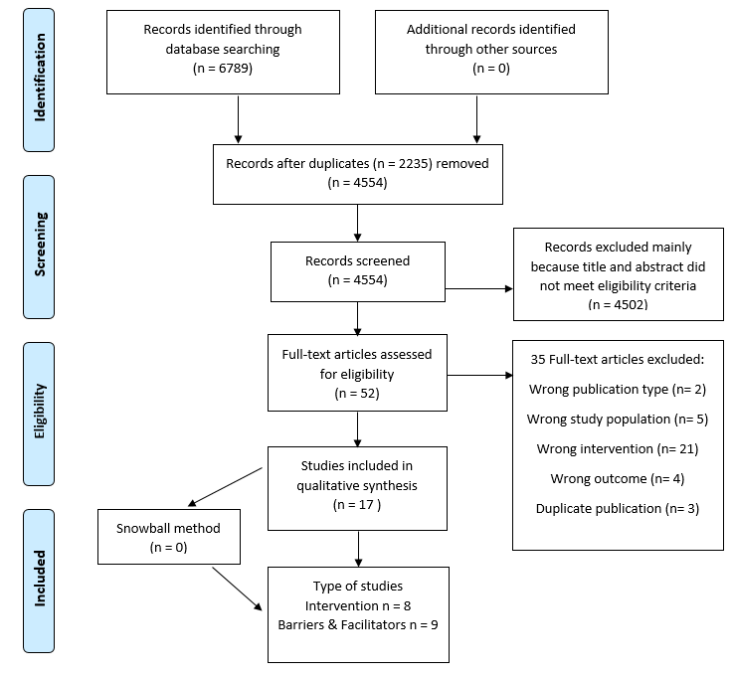
PRISMA flowchart.

The initial search generated 6789 articles. After removal of duplicates and initial screening of titles and abstracts, 52 studies remained for full-text assessment. Of these 52 studies, 17 articles met our eligibility criteria and were included in this systematic review. Figure 2 depicts the PRISMA flowchart for the study screening process.

### Study Characteristics

#### Overview

Of the 17 included studies, eight were clinical studies that implemented a digital care platform and assessed its effect on at least one of the predefined outcome parameters [37-44]. Four of these clinical studies [39,42-44] also described barriers and facilitators for implementation. The other nine studies [45-53] only investigated barriers and facilitators for digital care platforms. None of the included studies was written in a non-English language. Table 1 and Table 2 show the baseline characteristics of the clinical studies and the barrier and facilitator studies included in this review, respectively.

**Table 1:** Baseline characteristics of clinical studies.

Reference	Country	Study design	Patient population and sample size	Platform studied	Features of digital care platform				Outcome parameters					Q1^a^		Q2^b^
					General information	Access to EMR^c^	e-Consult^d^	Availability of information		Self-efficacy	COC^e^	PRE^f^/ HCPE^g^				
Baker et al [37]	US	RCT^h^	450 patients with BCA^i^	CHESS^j^	✔	X	✔	✔		✔	X	✔	✔		X	
Børøsund et al [38]	Norway	RCT	167 patients with BCA (<12 months)	Web-choice	✔	X	✔	X		✔	X	X	✔		X	
De Regge et al [39]	Belgium	Mixed methods	23 patients with metastatic kidney or bone cancer	DOP	✔	✔	✔	✔		X	X	✔	✔		✔	
Groen et al [40]	Netherlands	Feasibility study	37 patients with NSCLC^k^ (<12 months)	MyAVL	✔	✔	X	✔		✔	X	✔	✔		X	
Gustafson et al [41]	US	RCT	257 patients with BCA	CHESS	✔	X	✔	X		✔	X	X	✔		X	
Kuijpers et al [42]	Netherlands	Mixed methods	92 patients with BCA (<12 months treatment)	MyAVL	✔	✔	X	✔		✔	X	✔	✔		✔	
Ruland et al [43]	Norway	Prospective cohort	103 patients with BCA (n=56) and PCA^l^ (n=47)	Web-choice	✔	X	✔	✔		X	✔	✔	✔		✔	
Tiong et al [44]	Australia	Prospective pilot study	50 patients with BCA	Healthy.me	✔	X	✔	✔		✔	✔	✔	✔		✔	

^a^Q1: addresses research question 1 (to collect the best available evidence of the effect of a digital care platform on quality of care for oncological patients).

^b^Q2: addresses research question 2 (to analyze the currently reported barriers and facilitators for implementation of a digital platform in oncological health care).

^c^EMR: electronic medical record.

^d^e-Consult: electronic consult.

^e^COC: continuity of care.

^f^PRE: patient-reported experiences.

^g^HCPE: health care provider–reported experiences.

^h^RCT: randomized controlled trial.

^i^BCA: breast cancer.

^j^CHESS: Comprehensive Health Enhancement and Support System.

^k^NSCLC: nonsmall cell lung cancer.

^l^PCA: prostate cancer.

**Table 2:** Baseline characteristics of barrier and facilitator studies.

Reference	Country	Study design	Patient population and sample size	Description of platform	Q1^a^	Q2^b^
Alpert et al [45]	US	In-depth semistructured interviews with cancer patients and oncologists	35 cancer patients (breast n=9, hematologic n=6, gastrointestinal n=5, genitourinary n=4, lung n=3, sarcoma n=3, skin n=3, gynecologic n=2, other n=3) and 13 oncologists	Patient portals offer functional benefits to patients as they enable convenient patient access to EMR^c^ data from devices such as personal computers or smartphones; they allow the ability to request medication refills, schedule appointments, and they support secure messaging	X	✔
Baudendistel et al [46]	Germany	Qualitative explorative study using focus groups	12 colorectal cancer patients, 17 physicians, and 26 other health care professionals	Patient-controlled “personal electronic health record” (PEPA) (in development)	X	✔
Ector et al [47]	Netherlands	Design thinking development study	8 CML^d^ patients, 14 hematologists	CMyLife, a web-based, patient-centered intervention	X	✔
Geerts et al [48]	Netherlands	Mixed methods design: focus group and literature for patient survey, and physician survey based on literature	Patients with hematologic malignancy (questionnaire: n=204, focus group: n=6) and their physicians (questionnaire: n=13)	Not yet developed	X	✔
Gerber et al [49]	US	Qualitative study using 2 focus groups	13 nurses from a cancer center	Not yet developed	X	✔
Girault et al [50]	Canada	Questionnaire-based survey	1072 patients treated in a comprehensive cancer center	Internet-based technologies such as patient portals, websites, and applications managed by health care institutions to improve cancer care coordination	X	✔
Kildea et al [51]	Canada	Participatory stakeholder design	Focus group and survey among 361 cancer patients	Electronic patient portal accessible through smartphones (not yet developed)	X	✔
Kuijpers et al [52]	Netherlands	Focus group interviewing (9 groups)	21 BCA^e^ patients, 31 lung cancer patients, and 31 health professionals	Interactive portal	X	✔
McCleary et al [53]	US	Focus group sessions and surveys	Focus group: 20 patients and family and 5 advisory council members; survey: 1019 cancer patients	Patient gateway application as patient portal: a secure, web-based database enabling patient access to their health and disease information embedded within an EMR, managed by individual health care organizations and accessible via the internet	X	✔
De Regge et al [39]	Belgium	Mixed method triangulation design	Interviews with 23 patients, 2 physicians, 1 nurse specialist, 2 nurse consultants, 9 nurses, and 1 psychologist	The digital oncology platform includes the individualized care path, reliable treatment-related information, contact details for the treatment team, secure conversations with the treatment team, self-registration of complaints, and a diary	✔	✔
Kuijpers et al [42]	Netherlands	Mixed methods design: pretest-posttest design and focus group	6/92 BCA patients were included in focus group discussions; 24 health care providers were asked to fill out a questionnaire	MyAvL includes personalized educational material, overview of past and upcoming appointments, EMRs, medication overview, questionnaire concerning PROMs^f^	✔	✔
Ruland et al [43]	Norway	Prospective cohort	103 patients with breast cancer (n=56) and prostate cancer (n=47) received questionnaires with space for additional comments and suggestions	WebChoice is an interactive electronic health application that includes personalized information, a communication tool where patients can receive support from peers or professionals, a diary, and a self-management component	✔	✔
Tiong et al [44]	Australia	Prospective pilot study	50 patients with BCA were asked to use the platform and fill out a questionnaire; 9 patients were invited for a face-to-face feedback session	Healthy.me is a secure personally controlled health management website that features the patient journey with tailored information, appointment overviews, interactive forums, and messaging with peers and private messaging with health care providers	✔	✔

^a^Q1: addresses research question 1 (to collect the best available evidence of the effect of a digital care platform on quality of care for oncological patients).

^b^Q2: addresses research question 2 (to analyze the currently reported barriers and facilitators for implementation of a digital platform in oncological health care).

^c^EMR: electronic medical record.

^d^CML: chronic myeloid leukemia.

^e^BCA: breast cancer.

^f^PROM: patient-reported outcome measure.

#### Study Design

Among the clinical studies, three were RCTs [37,38,41], two were mixed methods studies [39,42], and three were pilot or feasibility studies [40,43,44] investigating the use of the platform in a small cohort. The three RCTs included 450, 167, and 257 patients, respectively, and all assessed the effectiveness of different variations of a digital care platform [37,38,41]. For example, Baker et al [37] assessed the effectiveness of different components of a digital care platform by comparing patient groups using a platform with information only; a platform with information and support; and a platform with information, support, and coaching. The control group did not use a digital care platform but used the internet. An example of one of the mixed methods studies is that performed by Kuijpers et al [42], in which a digital care platform was implemented in a small cohort with a postintervention questionnaire, followed by focus group discussions. Studies solely investigating barriers and facilitators were mainly qualitative in nature, with the exception of three studies applying a mixed methods methodology [48,51,53] and one study using a quantitative methodology [50].

#### Patient Population

Breast cancer patients formed the majority of the study population of the clinical studies, with 75% of all studies including solely this patient population. The patient population was therefore mostly female, highly educated, and young (mean age 50 years) [37,38,42,43]. The other studies included patients with nonsmall cell lung cancer or a diverse group a of cancer patients such as those with renal cell cancer and sarcoma. One study [43] included two different patient groups, namely breast cancer and prostate cancer patients, and compared the use and effectiveness of the digital care platform between these two groups. The barrier and facilitators studies (Table 2) included patients with colorectal cancer, lung cancer, breast cancer, hematological cancer, and a variety of other cancer types.

#### Interventions

Within the eight clinical studies, five web-based platforms were distinguished. These platforms were all web-based and were not integrated in the electronic medical record (EMR). Seventy-five percent of the studies assessed a platform that also intended to improve self-management by, for instance, symptom monitoring, physical activity advice, or self-therapy [37,38,40-43]. Although each digital care platform had at least two of the three predefined criteria, heterogeneity concerning the platforms was observed. For example, the digital care platform described by De Regge et al [39] enabled HCPs, including general practitioners, to read their patients’ medical records from other health care centers. This allowed them to gain insight into their patients’ treatment trajectory and decision-making by other involved HCPs. Other platforms such as those described by Groen et al [40] and Kuijpers et al [42] did not have this feature, and provided personalized patient education material, an overview of upcoming hospital appointments, and tailored physical activity. 

### Methodological Quality

Quality assessment of the included studies is shown in Table 3. Of the clinical studies, four studies were of low quality [37,41,43,44], three of moderate quality [38,39,42], and one study was assessed as a high-quality study [40]. Regarding the barrier and facilitator studies, three studies were of low quality [49,51,53], one of moderate quality [48], and five were of high quality [45-47,50,52].

**Table 3:** Mixed Methods Appraisal Tool scoring of the included studies (N=17).

Reference		Qualitative						Quantitative RCTs^a^						Quantitative nonrandomized						Quantitative descriptive						Mixed methods^b^						Total score^c^
		1	2	3	4	5	1		2	3	4	5	1		2	3	4	5	1		2	3	4	5	1		2	3	4	5		
**Clinical studies**																																
	Baker et al [37]	—^d^	—	—	—	—	N^e^		?^f^	?	?	?	—		—	—	—	—	—		—	—	—	—	—		—	—	—	—	0	
	Børøsund et al [38]	—	—	—	—	—	?		Y^g^	Y	?	Y	—		—	—	—	—	—		—	—	—	—	—		—	—	—	—	3	
	Groen et al [40]	—	—	—	—	—	—		—	—	—	—	Y		Y	Y	?	Y	—		—	—	—	—	—		—	—	—	—	4	
	Gustafson et al [41]	—	—	—	—	—	1		?	?	?	?	—		—	—	—	—	—		—	—	—	—	—		—	—	—	—	1	
**Clinical studies with barriers and facilitators**																																
	de Regge et al [39]	Y	Y	?	N	Y	—		—	—	—	—	—		—	—	—	—	Y		Y	Y	Y	Y	Y		?	Y	Y	N	3	
	Kuijpers et al [42]	—	—	—	—	—	—		—	—	—	—	N		Y	N	Y	Y	—		—	—	—	—	—		—	—	—	—	3	
	Ruland et al [43]	—	—	—	—	—	—		—	—	—	—	Y		N	?	?	?	—		—	—	—	—	—		—	—	—	—	1	
	Tiong et al [44]	—	—	—	—	—	—		—	—	—	—	N		?	Y	N	?	—		—	—	—	—	—		—	—	—	—	1	
**Barrier and facilitator studies**																																
	Alpert et al [45]	Y	Y	Y	Y	Y	—		—	—	—	—	—		—	—	—	—	—		—	—	—	—	—		—	—	—	—	5	
	Baudendistel et al [46]	Y	Y	Y	Y	Y	—		—	—	—	—	—		—	—	—	—	—		—	—	—	—	—		—	—	—	—	5	
	Ector et al [47]	Y	Y	Y	Y	Y	—		—	—	—	—	—		—	—	—	—	—		—	—	—	—	—		—	—	—	—	5	
	Geerts et al [48]	Y	Y	?	N	?	—		—	—	—	—	—		—	—	—	—	Y		Y	Y	?	Y	Y		Y	Y	?	N	3	
	Gerber et al [49]	Y	Y	?	?	N	—		—	—	—	—	—		—	—	—	—	—		—	—	—	—	—		—	—	—	—	2	
	Girault et al [50]	—	—	—	—	—	—		—	—	—	—	—		—	—	—	—	Y		Y	?	Y	Y	—		—	—	—	—	4	
	Kildea et al [51]	Y	Y	?	N	?	—		—	—	—	—	—		—	—	—	—	Y		Y	?	?	?	Y		?	?	?	N	1	
	Kuijpers et al [52]	Y	Y	?	Y	Y	—		—	—	—	—	—		—	—	—	—	—		—	—	—	—	—		—	—	—	—	4	
	McCleary et al [53]	Y	Y	?	N	?	—		—	—	—	—	—		—	—	—	—	Y		Y	?	Y	Y	?		N	?	Y	N	1	

^a^RCT: randomized controlled trial.

^b^For mixed methods studies, a score of 5.5 was required to be evaluated using category 1 and category 3 or 4.

^c^Total score: 0-2=low quality, 3=intermediate quality, 4-5=high quality.

^d^Not relevant.

^e^N: no.

^f^?: cannot tell.

^g^Y: yes.

### Outcome Measures

#### Enhancing Availability of Information

Five studies [39,40,42-44] reported on enhancing availability of general information on a digital care platform. In a high-quality feasibility study [40], the option to receive information was scored by patients with a mean of 7.1 (SD 1.5) on a scale of 1-10. The actual usage of information sections in the digital care platform differed among studies. One high-quality study reported the patient EMR to be the most used section of the digital care platform (mean 6.7, SD 4.7 logins during the 4-month study period) [40] and another study of moderate quality reported the opposite finding that the information section was rarely viewed (mean 0.75, SD 1.4 number of times participants consulted the information section) [39]. Needs of patients also differed, as Ruland et al [43] reported a significant difference (625 vs 271, *P*=.01) in the number of visits to the information section between breast cancer and prostate cancer patients, with breast cancer patients visiting this section more often. In this study, which was appraised as low quality, information comprehensibility of the digital care platform was rated with an overall mean score of 7.2 (SD 1.4) and usefulness was rated with a mean score of 6.5 (SD 1.7), both on a scale of 1-10 [43]. The main reasons for consulting the general disease-related information section of the digital care platform were to get help with problems and to get assurance. In addition to the information section, the platform’s ability to directly communicate with a nurse was perceived as very useful, because it was perceived as easy-to-understand information. The nurses’ contribution to a patient forum was reported as a trustworthy source of information [43] and therefore was highly valued by patients [44].

#### Self-Efficacy

Six studies investigated the effect of a digital care platform on self-efficacy [37,38,40-42,44] and all of them reported positive effects. Although the effects were not significant, a clear trend toward a positive effect on self-efficacy was reported. In the RCT performed by Børøsund et al [38], a moderate-quality study, patients using a digital care platform tended to score higher on self-efficacy compared with patients in the usual care group (mean difference 8.81, range 33-297, 95% CI –0.92 to 18.53, *P*=.08). Patients reported that a digital care platform was helpful in managing their health [44]. Groen et al [40] and Kuijpers et al [42], studies with high to intermediate quality, reported that access to general disease-related information and patients’ EMRs enhanced patients’ knowledge of their disease and their sense of control, based on questionnaires. Two RCTs showed that using a digital care platform increased patients’ perceived ability to obtain and use health care information. The digital care platform also increased their comfort and activation level of dealing with physicians and health care situations compared to the patient group only using the internet [37,41]. However, both of these RCTs were categorized as low quality.

#### Continuity of Care

None of the included clinical studies reported specifically on continuity of care as a primary or secondary outcome parameter. However, three low/intermediate-quality studies [38,39,43] reported on the aspect of communication between patients and HCPs and among HCPs within the platform. A questionnaire-based pilot study [43] reported that the most valued feature of the platform (ie, Webchoice) as expressed by the study population was the ability to send messages to their HCPs. That same research group investigated the effects of their platform in an RCT and reported 40% use of the messaging service in this platform [38]. Patients perceived this feature to be useful and easy to understand. De Regge et al [39] reported that HCPs perceived the ability to exchange research results between HCPs (eg, HCPs in other hospitals and primary care) as valuable to patient care and a means to optimize continuity of care across institutes.

#### Patient- and HCP-Reported Experiences

Seven studies [37-40,42-44] reported on patient- or HCP-reported experiences. Patient satisfaction with the studied platform was considerably high in three intermediate/high-quality studies, with a mean rating of 3.9 (range 3.8-4.09) on a 1-5 scale [39,40,42]. A low-quality RCT that aimed to compare the effects of three different types of interventions and one control observed significantly higher patient satisfaction scores with their HCPs compared with those of the control group at 6 weeks (3.46 vs 3.17, *P*=.01) and 6 months (3.48 vs 3.28, *P*=.03) [37]. The majority of patients (75%-93%) stated that the platform was easy to use and that it was a valuable addition to their health care experience [40,42]. The most used features of the platform varied among studies, but mainly consisted of the personal medical records in intermediate/high-quality studies [39,40,42]. Only two clinical studies reported the experiences by HCPs. De Regge et al [39], an intermediate-quality study, reported that HCPs perceived the digital care platform as valuable compared to current care because it provides reliable, easy-to-access information for patients and because it enables the exchange of patient-related information between care providers. Despite these positive experiences, one-quarter of the physicians interviewed by Kuijpers et al [42], an intermediate-quality study, reported an increase in workload after installment of the digital care platform, varying from a few extra minutes to more than 10 minutes per patient, for additional explanations on information made available on the platform.

### Barriers and Facilitators Levels

#### Overview

**Figure 3:**
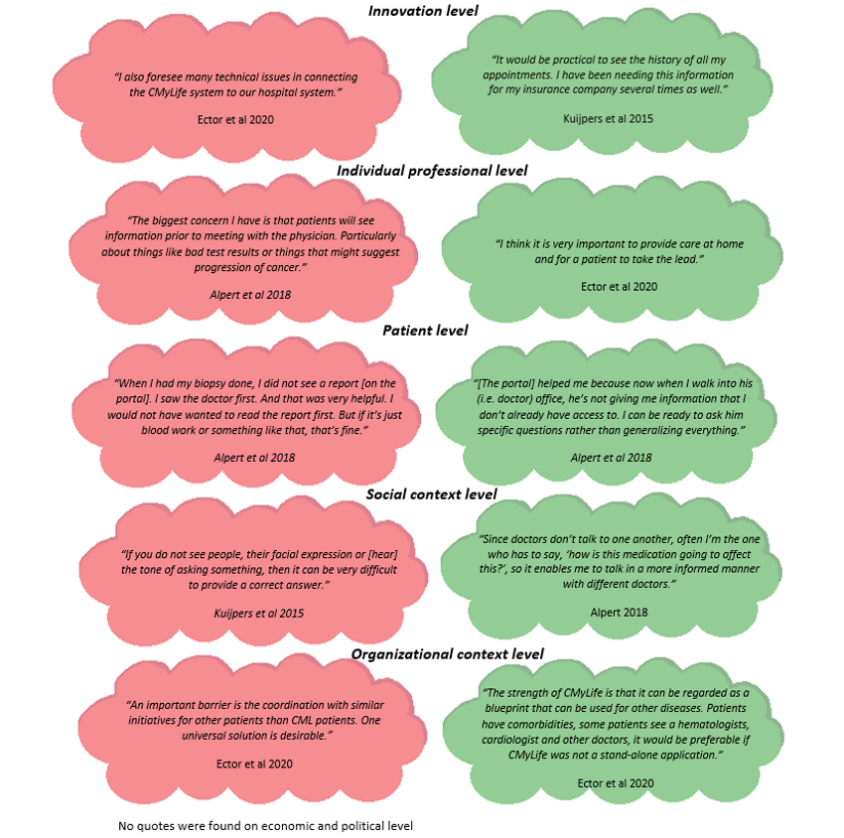
Illustrative quotations mentioned in included studies concerning barriers (in red) and facilitators (in green) for implementation of a digital platform.

Table 2 presents the baseline characteristics of the studies describing barriers and facilitators. Tables S1 and S2 in Multimedia Appendix 1 present all barriers and facilitators identified in the included studies. The most prominent barriers and facilitators are discussed below. Figure 3 depicts the illustrative quotations found in the studies for each level.

#### Innovation Level

Four studies [44,46,48,51] described technical challenges as a possible barrier for implementation. These included software development, functionality of the website, bugs, and log-on and internet access issues. A second barrier, concerning accessibility of the innovation, was patients’ unawareness of the existence of the digital care platform, which led to limited or no use of the platform [53]. Despite these barriers, three studies showed that the use of a digital care platform is feasible [40,42,44]. Facilitators in practice were numerous: access to general and tailored medical information [42,45,47-49,52], full EMRs [39,42,45-48,50-52], and educational materials [42,47]. Moreover, the possibility to consult HCPs through interactive tools was mentioned to be a positive feature of a digital care platform that encouraged usage [43,44,47,48,50]. 

#### Individual Professional Level

The most frequently described barrier at the professional level was the concern of HCPs regarding the release of medical information on the digital care platform and the comprehensibility of medical jargon for patients [39,45,46,48,49,51,52]. Patients learning about a change in their health and well-being via a digital care platform was a fear expressed by all oncologists included in two studies [45,48]. The second most frequently described barrier, described in seven studies [39,42,45-47,49,51], concerned the fear of increased workload such as an increased burden of documentation, monitoring, and direct digital communication with patients [39,42,45,49].

Concerning facilitators, one study suggested that a digital care platform would not increase workload but would rather reduce workload by easing data management [46]. Another facilitator mentioned was the ability to exchange information or medical results between different HCPs, including those in primary care such as general practitioners [39,46]. The optimization of data exchange between institutes could avoid or reduce repeated health assessments [46].

#### Patient Level

Three studies indicated that patients had concerns about the readability of the information (medical jargon) displayed on the digital care platform and whether medical results could be adequately understood without professional interpretation [45,46,48]. Some patients mentioned that this could increase anxiety [48,52].

Facilitators at the patient level were numerous, as indicated in Table S2 of Multimedia Appendix 1. In several studies, patients and oncologists mentioned that patients having access to the platform and their EMR could better prepare them for their doctor visit and enabled them to take on an active role during the consultation [45-47]. In addition, patients’ willingness to communicate via digital resources facilitated implementation [53]. Lastly, improved patient-doctor communication was described as a facilitator, either by improved preparedness for doctor visits or by direct messaging [52].

#### Social Context Level

Three studies reported concerns on how a digital care platform could change the doctor-patient relationship [48] such as by increasing the patient’s autonomous handling of information [46]. The digital care platform could make patients increasingly reliant on technology with decreased reliance on face-to-face communication [46,49]. This was feared as it may negatively influence the outpatient clinic visits as patients become more focused on discussing (irrelevant) details regarding available biomedical results rather than discussing values and preferences important for a follow-up treatment [48].

The change of the doctor-patient relationship was also mentioned as a facilitator. By enabling access to medical information, patients could become more actively involved in the management of their care and feel more comfortable to interact with their HCPs during consultations [42,45].

#### Organizational Context Level

Two studies mentioned the integration of the platform into existing systems as a barrier [44,47]. This would be especially relevant in cases in which the digital care platforms were also developed for other chronic diseases [47]. Limitations in time and resources were reported as another type of barrier [44].

A clear facilitator at the organizational level was the digital care platform’s ability to exchange important patient information across health care institutes. HCPs emphasized the advantage of a digital care platform in improving the cross-sectoral availability of information about the patient to HCPs who are involved in the patient’s treatment. It is suggested that the digital care platform could thereby diminish information loss, be of use in emergency situations where the rapid release of medical information is vital, and avoid repeated diagnostic investigations [46]. Organization of care could be improved as the improved availability of information promotes cross-boundary continuity of care and diminishes fragmentation of care [39,46]. Another facilitator at the organizational level was the early introduction of the digital care platform to the patient, preferably at diagnosis [44], and sufficient instructions concerning appropriate use of the platform for patients as well as HCPs [49].

#### Economic and Political Contexts Level

Data security and protection regarding the digital care platform were concerns reported by numerous studies [46,50,51,53]. During the development of the digital care platform described by Kildea et al [51], cybersecurity testing and legal issues were the most time-consuming processes. These legal issues involved the ownership of intellectual property [51] and liability [46,51].

Secure access was therefore identified as an important facilitator for a digital care platform [48]. Another facilitator, in the economical context, was that conferred by the optimized exchange of patient information between institutes, enabled with a digital care platform, and that unnecessary repeated diagnostic procedures and health assessments could be avoided [46].

## Discussion

### Principal Findings

A digital care platform for oncological patients has the potential to improve quality of care through the improved availability of information and positive effect on self-efficacy. Although continuity of care was not studied as a primary outcome in the identified studies, based on focus group interviews with HCPs, a digital care platform potentially improves continuity of care by optimizing the exchange of patient information across institutes. Patient-reported experiences such as satisfaction with the platforms were considerably positive. Our barrier and facilitator analysis indicated that the majority of barriers exist at the professional level. This included a concern of increased workload for HCPs and release of unattended medical information to patients. The majority of facilitators were identified at the patient and innovation levels. The patient’s ability to become more informed, empowered, and involved in their care was identified as a prominent facilitator. Another relevant facilitator, at the organization level, is the digital care platform’s potential to improve information exchange between HCPs across different institutes. This is relevant as this may improve continuity of care and diminish fragmentation of oncological care. Among the clinical studies, the majority were of low to intermediate quality. Regarding the barrier and facilitator studies, the majority were of high quality.

The positive effects of a digital care platform described in this review are consistent with prior research studying isolated features of digital care platforms [16,29,54]. A digital care platform seems to be a helpful medium in providing patients with general disease-related and personal information. This is a welcoming result as previous studies have described that a considerable number of patients are dissatisfied with information provision [55,56]. Moreover, 40%-80% of all medical information provided by the HCP during a consultation is forgotten or remembered incorrectly [57]. Therefore, the need of patients to read or reread medical information that is relevant for their situation is an important facilitator for a digital care platform. In this systematic review, we identified studies that showed positive effects on self-efficacy, but no convincing clinically significant effects. In another systematic review, which studied the effectiveness of eHealth-based self-management tools, significant yet small effects on self-efficacy were described [25]. The authors explain that the tool was able to increase self-efficacy by enabling patients to enhance participation in their care trajectory. A more recent randomized trial refuted this finding [58]. In this RCT, the effect of an eHealth-based self-management tool (Oncokompas) was investigated in 600 cancer patients. At 3 and 6 months, patients were assessed on self-efficacy with the General Self-Efficacy scale. No significant effects on self-efficacy were observed (mean difference 0.5, 95% CI –0.4 to 1.4, *P*=.31). The authors partially attributed the lack of effect to the included patient population, which mostly consisted of patients diagnosed with cancer 2 years prior to the study. The authors suggested that this population is perhaps less in need of a self-management tool to increase self-efficacy as they have more experience and “know-how” compared with their newly diagnosed peers. The hypothesis that the effect of an eHealth-based tool may vary considerably depending on the patient population is important to consider for assessing the effectiveness of a digital care platform. In our systematic review, we included studies with patients in different stages of their disease trajectory and with different cancer types, and all effects regarding self-efficacy were positive but not significant. On the one hand, this aspect can be perceived as a limitation because the heterogeneity of patients may underestimate the effect of a digital care platform. On the other hand, it is a strength as we now have an overview of the effect of a digital care platform on self-efficacy of a broad cancer patient population, thereby increasing the generalizability of our results.

Similarity with prior work concerns the limited number of studies investigating combined features of a digital care platform. During the screening process of this review, we encountered numerous studies that only investigated one feature of the platform. This finding is similar to the findings of a review by Kruse et al [20], which concluded that many studies assessed the effect of one feature of a digital care platform and that a full platform was rarely studied in clinical practice. To adequately ascribe outcomes as an effect of the intervention, it is reasonable to first study an isolated feature of a digital care platform. However, it is absolutely vital to also study the combined features in a digital care platform for two key reasons: (1) because this is likely to be the eHealth intervention that is implemented in practice, and (2) because the effect of multiple features may not be equivalent to the sum of effects of a single feature.

The fact that we assessed the effects of digital care platforms that had at least two key features can therefore be considered a strength of this review. In addition, we assessed the effect of a digital care platform on quality of care parameters such as continuity of care, and described the patient- and HCP-related experiences. Other reviews did not, and instead only assessed the effect of a digital care platform with one feature or chose to study different endpoints such as fatigue, physical activity, depression, quality of life, and self-management abilities [20,25,29,30,54,59]. An additional strength is that we analyzed barriers and facilitators for implementation. This allows for a good understanding of what is needed for successful implementation. A final strength is that the search query was quite elaborate without exclusion of non-English articles, thereby minimizing the possibility of missing valuable studies. For these reasons, this systematic review provides a comprehensive overview of the best available evidence of the effect of a digital care platform on quality of care for oncological patients.

However, this systematic review also has some limitations. First, the included clinical studies were mostly early-stage, single-arm prospective studies where feasibility and acceptability were investigated. Although we did include three studies with a more advanced research methodology (ie, RCTs), these studies were all appraised as studies with low to intermediate quality. This makes interpretation of these RCTs quite challenging. One RCT reported a significant difference in patient satisfaction scores favoring a type of digital care platform [37]; however, the minimal difference (3.48 vs 3.28 on a 1-5 scale) puts into question the clinical relevance of this finding. A second limitation of this review concerns the heterogeneity of study designs and of the digital care platforms. Despite our predefined criteria of what a digital care platform should include, they were still quite different from each other with respect to the specific web format, options, and intended use, among other aspects. This heterogeneity precluded a pooled analysis with quantitatively measured outcome parameters. A third limitation concerns the study population and its representativeness. Three-quarters of the clinical studies included breast cancer patients. This study population mainly consists of well-educated females [60]. Indeed, 72%-73% of the breast cancer population in the studies included in this review had a college or university degree. It is plausible that a high education is an important factor for successful use of a digital care platform. For this reason, results of this review should be interpreted with caution and cannot simply be generalized to other cancer patients. A final limitation concerns the barrier and facilitator analysis. Studies included in this review almost exclusively focused on the barriers and facilitators mentioned by the end users of the platform (ie, the patients and HCPs). Evidently, their perspectives are crucial, but barriers and facilitators proposed by other stakeholders should also be investigated, such as stakeholders with an organizational, economic, and political background (eg, health insurers). This latter group can provide insight into what is required to realize the structural financing of a digital care platform.

Although there is a growing body of literature that describes the positive effects of digital care platforms, high-quality studies describing the effectiveness of these platforms integrated in oncological care are currently lacking. In addition, most studies implemented a digital care platform in one target patient population such as breast cancer patients. More evidence is required concerning the desirability and use of a platform in patients with other types of cancers and education levels. More specifically, it is important to investigate whether patients with a lower level of education can benefit from digital care platforms to the same extent as their well-educated peers to avoid widened health disparities. The same applies to the patient population with limited internet access or internet skills. Studies in this review were performed in Australia, North America, and western Europe. The usefulness of digital care platforms in countries with low internet access is likely very different.

Concerning barriers and facilitators, future studies should aim to further elucidate barriers and facilitators at the organizational level and the economic and political levels. Ultimately, this is required for a digital care platform to become a successful eHealth-based tool in the improvement of quality of care for patients living with cancer.

### Conclusion

Digital care platforms have a favorable effect on availability of information and enhancement of self-efficacy. Additionally, they could potentially serve as a valuable medium to improve continuity of care by optimizing communication between patients and HCPs and among HCPs. The vast majority of patients are positive about a digital care platform and its ability to meet their needs in improving the availability of information and patient involvement. Although these results are favorable, they were mostly generated by early-stage, nonrandomized studies with a specific patient population. To fully understand whether a digital care platform is able to increase quality of care by supporting the delivery of coordinated, patient-centered oncological care, more advanced studies such as RCTs are required, as well as studies investigating the barriers and facilitators at the economic and political levels.

## Appendix

Multimedia Appendix 1 Tables S1 and S2.

## Abbreviations

e-Consult: electronic consultation
eHealth: electronic health
EMR: electronic medical record
HCP: health care professional
MMAT: Mixed Methods Appraisal Tool
PRISMA: Preferred Reporting Items for Systematic Reviews and Meta-Analyses
PROM: patient-reported outcome measure
PROSPERO: Prospective Register of Systematic Reviews
RCT: randomized controlled trial
