# Supplementary material for: Effect of Digital Care Platforms on Quality of Care for Oncological Patients and Barriers and Facilitators for Their Implementation: Systematic Review
Source: J Med Internet Res. 2021 Sep 24;23(9):e28869. doi: 10.2196/28869 (PMC8501408; doi:10.2196/28869)
Supplement: Multimedia Appendix 1 [file jmir_v23i9e28869_app1.docx]

# Table S1. Overview of barriers of digital care platform extracted from the included articles

| Level | **Innovation** | **Individual professional** | **Patient** | **Societal** | **Organization** | **Economic and political** |
| --- | --- | --- | --- | --- | --- | --- |
| Arguments | Technical challenges [38,40,42,45]  Not being aware of the availability of the DCP or its features [47] | Concerns about patients accessing medical results (or bad news) before meeting their HCP  [33,39,40,42,43,45,46]  Fear of increased workload [33,36,39-41,43,45] Patients would anticipate rapid communication [39,43]  Resistance to change by healthcare providers [39,42]  Concern of changing doctor-patient relationship [42]  Lack awareness of the features of the DCP [39]  Patients get insight in what physicians write about them [42]  Medical jargon could be a problem [36,39]  HCPs do not agree to write in plain language [42]  Differences in workflow and handling the DCP communications [43] | Patients not understanding medical information or medical jargon [36,39,40,42,43]  Concerns about patients seeing their test results before the appointment with their physician without further explanation by the physician [39,40,42]  Feeling obliged to prepare the appointment by studying portal information [42]  Need for commitment from patients for continuation of usage [38]  Differences in needs of new patients vs patients longer familiar with their disease [33,37] | Concern of decrease in direct communication between patient and physician [40,42,43]  Potential to widen health disparities [39] | Integration of platform in current care and other platforms  [38,41]  Limitations in time and resources [38]  Generational differences in dealing with data protection [40]  Lack of instruction on how to use the DCP [39,47] | Data protection, security and confidentiality and data security [40,44,45,47]  Legal aspect such as ownership of intellectual property, liability, contents of the patient disclaimer form [40,45] |
| DCP = digital care platform, EMR = electronic medical record | | | | | |  |

# Table S2. Overview of facilitators of digital care platform extracted from the included articles

| Level | **Innovation** | **Individual professional** | **Patient** | **Social context** | **Organizational context** | **Economic and political context** | |
| --- | --- | --- | --- | --- | --- | --- | --- |
| Arguments | Feasibility [34,35,38]  Visually attractive platform [36]  Educational materials [36,41]  Access to information and being able to re-read information [36,39,41-43,46]  Possibility to make and change appointments online [36,38,42]  Reminder for appointments [42]  Access to the full EMR [33,36,39-42,44-46] with easy-to-understand explanation [41]  Forum with peer support [37,38]  Communication with HCP (video consulting or messaging) [37,38,41,42,44]  Easy access through mobile application [33,38]  Improving medication management [40]  Overview of medication list [43] and medication history with the possibility to request medication recipe [42]  Glossary of medical jargon [42]  Insight into the patient-reported experiences and outcomes [41]  Better support in understanding and coping with the symptoms [41]  Improvement of guideline adherence [41]  Providing care only when medically needed or when desired by the patient [41]  Blood samples drawn at home [41] | Reduced workload for professionals by easing data management [40]  Feedback and advise from the HCP [36]  HCPs encouraging use of the platform [36]  HCPs promoting forum activity [38]  Physicians using plain language instead of medical jargon [42]  Preferring electronic communication over telephone conversations, it allows nurses to discuss and research their responses before answering questions [43]  Ability to exchange research results between care providers (e.g. other hospitals and primary care) [33,40] to avoid or reduce repeated health assessments [40] | Patient empowerment [36,39,41,42,46]  High use of internet based technologies, mobile phone and computer [44]  Better preparation of consults and better collaboration with HCP (better patient empowerment) [39-41]  Patients at home have a high information need related to understanding and dealing with cancer-related problems [37]  A DCP could help organize their medical information [40]  Improved patient-doctor communication with a DCP because of availability of contact information of the HCPs [45] and time to directly send questions that were not asked during consult [46]  Rapid access to important patient information, especially helpful in emergency situations [40]  Willingness to communicate via computer, indicating a comfort level with  communication via a DCP [47]  Perceived sense of direct communication [39] | Patients feel more comfortable when interacting with their oncologist when they had access to the portal in advance of appointments [36,39]  Believed that more knowledge about their condition from viewing the portal, enabled them to collaborate better with the provider [39] | Optimization of patient information exchange between healthcare institutes increases continuity of care [33,40]  Minimizing loss of information and avoiding multiple data collection and medical tests [40]  Early introduction to the tool [38]  Frequent content updates [38]  Education regarding appropriate use and expectations of a portal [43] | Multiple data collections, unnecessary repeat of medical investigations and health assessments could be avoided [40]  Enhancing adherence could save healthcare costs [41]  Secure and protected access to patient data [42] | |
| DCP = digital care platform, EMR = electronic medical record | | | | | |  |  |
